# Locally Advanced Cutaneous Squamous Cell Carcinoma: Two Case Reports of Dramatic Responses to Sequential Cisplatin, Docetaxel and Radiotherapy

**DOI:** 10.4021/wjon328w

**Published:** 2011-08-24

**Authors:** Kristopher E. Dennis, Charmaine Kim-Sing, Janessa Laskin

**Affiliations:** a Radiation Oncology Program, British Columbia Cancer Agency, Vancouver, British Columbia, Canada; b Division of Medical Oncology, British Columbia Cancer Agency, Vancouver, British Columbia, Canada

**Keywords:** Chemotherapy, Cutaneous, Locally advanced, Radiotherapy, Skin neoplasms, Squamous cell carcinoma

## Abstract

Systemic therapy for cutaneous squamous cell carcinoma is typically reserved for rare cases of metastatic disease or lesions which cannot be adequately managed with local therapies. The relevant published literature is comprised only of case series and reports. Herein the authors present two cases of neglected locally advanced cutaneous squamous cell carcinoma not initially amenable to local therapies, which achieved dramatic partial responses to palliative Cisplatin and Docetaxel, facilitating sequential palliative radiotherapy. These reports add to the limited literature supporting the use of platinum-based therapy in locally advanced and metastatic cutaneous squamous cell carcinoma. They are the first describing the use of Cisplatin and Docetaxel in this setting.

## Introduction

Non-melanomatous skin cancers are the most common forms of human malignancy, with more than 1 million cases expected to be newly diagnosed in the United States in 2009 [1]. Approximately 20% of these are squamous cell carcinomas (SCCs) [2] which show more aggressive behavior than the more common basal cell carcinomas [3]. The majority of SCCs are small, localized, not associated with nodal or distant metastases and are treated successfully with surgery or radiotherapy [4] with cure rates ranging from 90 - 95% [2]. Systemic therapy is typically reserved for rare cases of metastatic disease, or for lesions which cannot be adequately managed with local therapies due to large size or extensive local invasion. The published literature guiding systemic therapy in this regard, however, is limited and comprised largely of small case series where the regimens employed are adapted from those used to treat SCCs in other disease sites such as the head and neck, lung and esophagus. Platinum-based regimens have been the most commonly described [5-9].

The authors here aim to contribute to the literature by reporting two cases of locally advanced and metastatic cutaneous SCC treated with sequential palliative chemotherapy and radiotherapy.

## Case Report

### Case 1

**Figure 1:**
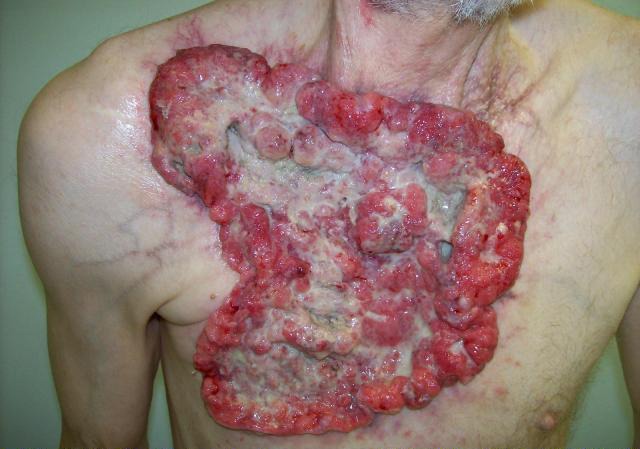
Locally advanced cutaneous squamous cell carcinoma of the chest wall.

**Figure 2:**
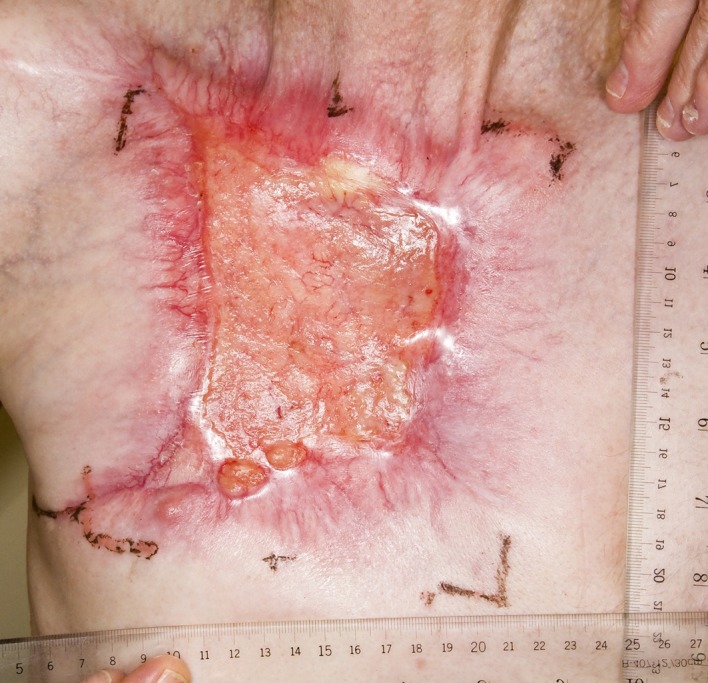
Partial response to palliative chemotherapy.

**Figure 3:**
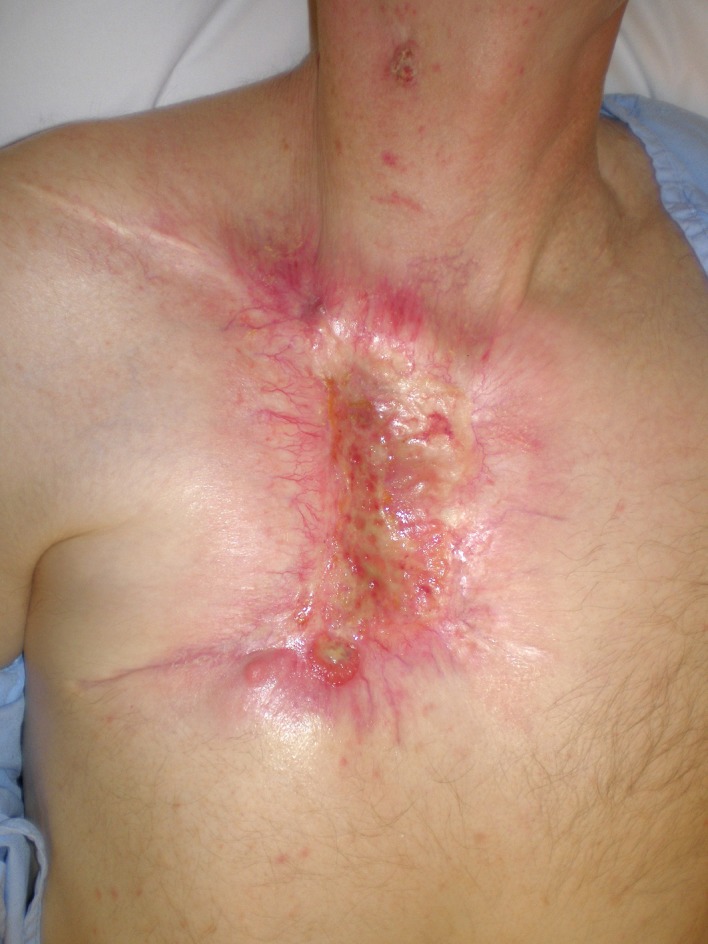
In-field recurrent chest wall nodules.

A 59-year-old Caucasian male presented with a 10-year history of a neglected slowly growing red lesion on the right anterior chest wall. Aside from tuberculosis treated in childhood and a remote right clavicle fracture he had no relevant past medical history, took no medications, and denied tobacco or alcohol consumption. He sought attention when the lesion’s growth rate increased and it began weeping serosanguinous fluid. He required transfusions for a presenting hemoglobin of 55 g/L. On examination there was a striking 20 x 15 cm non-odorous geographic lesion centered over the right chest wall, with heaped edges lying 3 cm proud of the normal skin contour and an ulcerated, weeping center (Fig. 1). It extended superiorly to the supraclavicular fossa, inferiorly below the inframammary fold, laterally to the anterior axillary line, and medially 5 cm beyond midline. In addition there was a 3 x 3 cm raised erythematous lesion suspicious for a satellite metastasis on the right forehead which had developed within the last year, and a long-standing unchanged 2 x 2 cm non-specific erythematous plaque on the left temple at the site of a remote chemical burn. Beyond chronic right shoulder achiness and motion restriction he denied pain, other local or constitutional symptoms and had an ECOG performance status of zero. CT from the chest to pelvis confirmed a large disc-shaped lesion invading the chest wall to a depth of 2 cm, destroying the right anterior manubrium and anteromedial right clavicle. There were calcified pulmonary nodules consistent with prior granulomatous disease, but no regional or distant lymphadenopathy and no visceral metastases. An incisional biopsy of the chest wall lesion confirmed moderately to poorly differentiated squamous cell carcinoma, and he was staged as pT4 cN0 cM1. The size of the lesion was prohibitively large for either surgery or radiotherapy so he proceeded to palliative chemotherapy as follows: Cisplatin 55 mg/m^2^ and Docetaxel 55 mg/m^2^ every 3 weeks. He tolerated 12 cycles extremely well with only mild myelosuppression, oral mucositis and tinnitus. The chest lesion showed an early and sustained dramatic response to therapy (Fig. 2). The right forehead and left temple lesions remained stable. He went on to receive palliative radiotherapy to the chest lesion as follows: 5000 cGy in 20 fractions via 9 MeV electrons to a 15 x 15 cm field, and to the right forehead lesion as follows: 4500 cGy in 15 fractions via 9 MeV electrons to a 7 x 9 cm field. Both lesions showed further improvement. He sustained a significant progression-free interval: 16 months from the time of initiation of chemotherapy and 6 months from the time of radiotherapy completion, but then developed multiple in-field recurrent chest nodules (Fig. 3) which were re-treated with 3000 cGy in 10 fractions via 9 MeV electrons to a 7 x 14 cm field. The long-standing left temple lesion had also begun to progress, and because of this it was deemed to indeed be malignant and was treated with 3500 cGy in 5 fractions via 9 MeV electrons to a 4 cm diameter circular field. At the time of manuscript preparation, 21 months following the time of initiation of chemotherapy, he had developed new satellite metastases on the neck and left shoulder, but was doing well overall with an ECOG performance status of zero.

### Case 2

**Figure 4:**
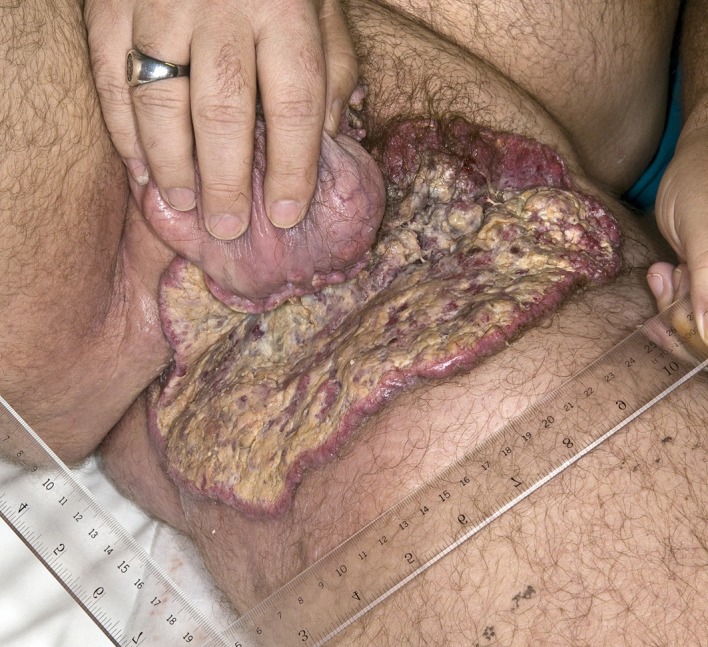
Locally advanced cutaneous squamous cell carcinoma of the left groin.

**Figure 5:**
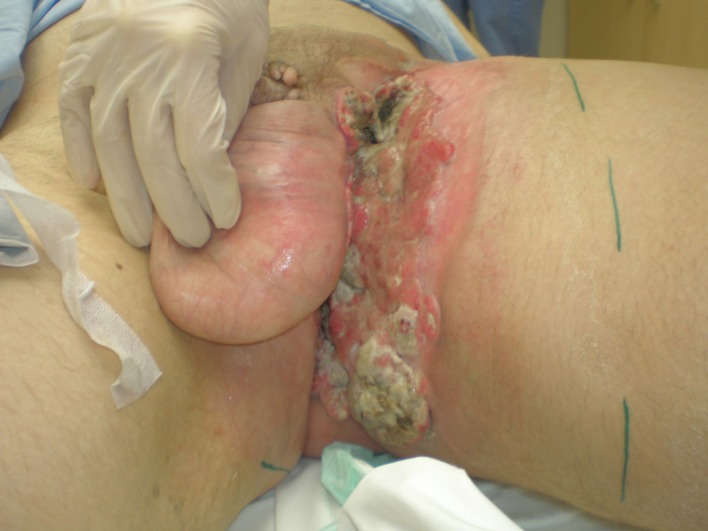
Partial response to palliative chemotherapy.

**Figure 6:**
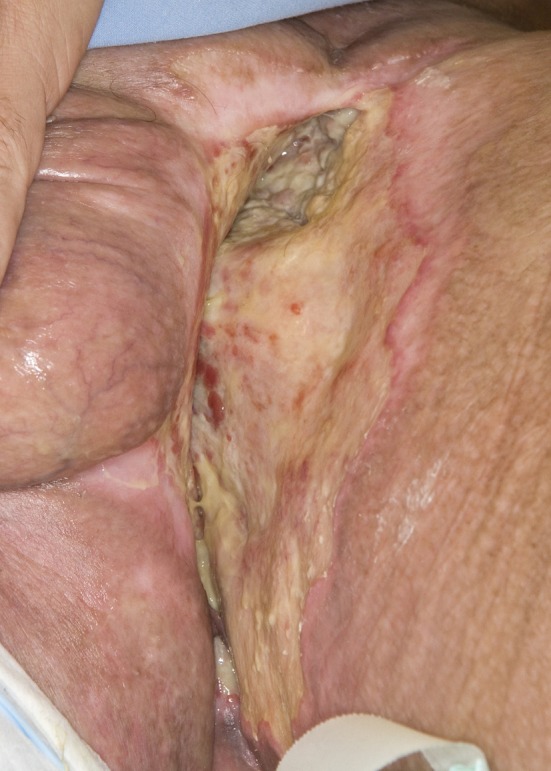
Further response to palliative radiotherapy.

A 46-year-old Caucasian male presented with a 6-month history of a neglected rapidly growing lesion on the proximal left thigh. He had no relevant past medical history and took no medications, but admitted to binge drinking on weekends and a 10 pack-year history of smoking. He sought attention when the lesion became painful and malodorous. On examination there was a large approximately 30 x 30 cm geographic lesion centered in the left groin, with heaped edges lying 2 cm proud of the normal skin contour, and an ulcerated raised heterogeneous center. It extended superiorly to the lower abdomen, inferiorly 10 cm below the base of the buttock, laterally to the lateral aspect of the inguinal crease, and medially to involve the scrotum, penile base and contralateral perineum (Fig. 4). In addition there was 2 x 2 cm pigmented polypoid lesion at the dorsal penile base. He had stiffness within the proximal left thigh, had lost 25 kg over the last 6 months and had an ECOG performance status of one. CT of the abdomen and pelvis confirmed a large plate-like lesion lying superficial to the adductor muscle group and femoral vessels, as well as left inguinal and external iliac lymphadenopathy up to 5.2 cm in greatest dimension. A chest x-ray was normal. An incisional biopsy confirmed moderately differentiated squamous cell carcinoma, and he was staged as cT3 cN1 cM1. His disease was prohibitively large for either surgery or radiotherapy so he proceeded to palliative chemotherapy as follows: Cisplatin 75 mg/m^2^ and Docetaxel 75 mg/m^2^ every 3 weeks. He tolerated 7 cycles; the first 2 at full dose strength and the last 5 at 75% dose strength (55 mg/m^2^) due to myalgias, oral mucositis and 2 episodes of febrile neutropenia requiring hospitalization and G-CSF support. The visible lesion showed an early and sustained significant response to therapy (Fig. 5), however, after 7 cycles CT documented progressive inguinal lymphadenopathy so he went on to receive palliative radiotherapy as follows: 5000 cGy in 25 fractions via an anterior/posterior parallel pair of 6 and 10 MV photons to a 29 cm (superior-inferior) x 38 cm (medial-lateral) field. The lesion showed further improvement (Fig. 6), however, CT 4 months following radiotherapy noted new left common iliac lymphadenopathy and CT 3 months later confirmed progression of the primary lesion as well, with increased pelvic lymphadenopathy and a non-occlusive tumor thrombus invading the left common iliac vein and IVC to the level of the renal veins. At the time of manuscript preparation, however, 15 months following the time of initiation of chemotherapy he surprisingly maintained an ECOG performance status of zero and was working full-time.

## Discussion

The responses induced by Cisplatin and Docetaxel in the present cases of locally advanced cutaneous SCC are similar to those described within other case series in the literature, the majority of which employed platinum-based regimens. When lesions are not amenable to initial surgery or radiotherapy, platinum-based regimens appear to offer the best chance of inducing responses that allow sequential consolidative local therapies.

Sadek et al. [5] reported a series including 13 evaluable patients with large cutaneous SCCs of the lip and skin treated with systemic therapy. Four patients had received prior surgery, 4 surgery and radiotherapy, and 1 chemotherapy. Nine had clinical nodal involvement, but all were free of distant metastatic disease. The authors implemented a protocol employed for other epidermoid carcinomas at their institute: Cisplatin 100 mg/m^2^ on day 1, 5-Fluorouracil 650 mg/m^2^/d by continuous infusion over 5 days, and Bleomycin 15 mg bolus on day 1 and continuous infusion 16 mg/m^2^/d over 5 days. Cycles were repeated every 3 - 4 weeks, and patients received between 1 - 4 cycles. Four of the 13 patients achieved a complete response (CR): 1 of these then had surgery alone, 2 had radiotherapy alone and 1 had both, resulting in 2 patients having no evidence of disease (NED) at 13 and 22 months post-chemotherapy respectively, 1 having NED after further surgery and radiotherapy required for a local recurrence at 8 months post-chemotherapy, and 1 dying of disease at 19 months post-chemotherapy. Seven of the 13 patients achieved a partial response (PR): 2 of these then received no local therapy, 3 had radiotherapy alone, and 2 had surgery and radiotherapy, resulting in 4 patients dying of their disease at 12, 11, 8 and 4 months post-chemotherapy respectively, and 3 having NED after 12, 11 and 10 months post-chemotherapy respectively. One of the 13 patients had no response to chemotherapy, received subsequent interferon and died of disease 10 months post-chemotherapy. The last of the 13 patients progressed through chemotherapy, received subsequent retinoids and died of disease 4 months post-chemotherapy. Overall then, of 13 evaluable patients, 6 had NED and 7 had died of their disease at the time of manuscript preparation.

Denic [6] reported on 3 patients treated with systemic therapy for cutaneous SCCs of the head and neck region that were either unresectable or would have resulted in a major esthetic defect or functional loss with immediate surgery or radiotherapy. The first patient was a 13-year-old male with xeroderma pigmentosum who had received previous surgery for a SCC of the orbit, the second patient also had xeroderma pigmentosum and had received previous radiotherapy for a SCC on the nose, and the third patient had received previous radiotherapy for a SCC on the lip. All patients received Cisplatin 20 mg/m^2^/d for 4 days and Bleomycin 20 mg/d for 4 days via continuous infusion. The first patient received a reduced dose of Bleomycin (15 mg/d). Cycles were repeated every 3 weeks. The first patient achieved a CR, the second a PR, and the third progressed through chemotherapy. All underwent subsequent surgery within 3 weeks. No recurrences had been recorded during short follow-up periods of between 2 - 9 months.

Guthrie et al. [7] also reported on 12 patients with cutaneous SCC who were selected for Cisplatin-based therapy for lesions unamenable to local therapy. Patients received Cisplatin 75 mg/m^2^ and Doxorubicin 50 mg/m^2^ every 3 weeks for up to 4 cycles. Seven of the 12 patients received chemotherapy alone; 2 of these 7 achieved a CR and had NED at 12 and 4 months from the initiation of chemotherapy respectively, 2 achieved a PR (1 for 3 months before dying of progressive disease and 1 for 6 months before developing progressive disease), and 3 patients had no response to chemotherapy (2 were alive with progressive disease and one dead of progressive disease at the time of manuscript preparation). One of the 12 patients received chemotherapy followed by surgery which resulted in a PR after chemotherapy and a CR of 11 months’ duration following surgery. The remaining 4 of 12 patients received chemotherapy followed by radiotherapy: 2 had no response to chemotherapy but achieved CRs of 32 and 30 months post-radiotherapy respectively, 1 achieved a CR after chemotherapy that persisted for 13 months post-radiotherapy and 1 achieved a CR after chemotherapy that persisted for 11 months post-radiotherapy until that patient died of comorbid lung cancer.

Khansur and Kennedy [8] reported on 2 patients with metastatic cutaneous SCC and 5 patients with locally advanced cutaneous SCC. All had declined surgery and thus received Cisplatin 100 mg/m^2^ on day 1 and 5-Fluorouracil 1 g/m^2^/d via 4-day continuous infusion. Cycles were repeated every 3 weeks. Patients received between 2 - 6 cycles. The first patient with metastatic disease achieved a CR and had NED 24 months following chemotherapy. The second patient achieved a PR and developed progressive disease 3 months following chemotherapy. Two patients with locally advanced disease achieved CRs; one was lost to follow-up 3 months following chemotherapy, and the other died of a myocardial infarction but had NED 13 months following chemotherapy. Two patients with locally advanced disease achieved PRs; one achieved a subsequent CR following consolidative surgery and the other refused chemotherapy after 2 cycles and outcome data was not provided. One patient with locally advanced disease had no response to 6 cycles of chemotherapy and died of extensive locoregional disease and a CVA.

Other options for systemic treatment include retinoids and interferon-alpha. Lippman et al. [10] reported on 28 patients with cutaneous SCCs that had either failed previous therapy, were unamenable to local therapies, or were metastatic. The combination of 13-cis-Retinoic Acid (1 mg/kg/d) and subcutaneous recombinant human interferon-α-2a (3 million units/d) for 2 months produced a 93% response rate for locally advanced lesions and a 25% rate for patients with distant metastatic disease. Other reports describe responses to targeted therapies. Bauman et al. [11] reported on 2 elderly patients; the first had received previous surgery and radiotherapy for a locally advanced SCC of the scalp and achieved a CR with palliative Cetuximab, and the second had received previous radiotherapy and surgery for a SCC of the columnella and achieved a PR with palliative Cetuximab. Similarly, Suen et al. [12] reported on a heavily pre-treated elderly patient with a recurrent cutaneous SCC of the sternum who achieved a CR with 7 weekly cycles of palliative Cetuximab (250 mg/m^2^/week). The patient recurred one month later, but achieved a second CR after 7 additional cycles before developing progressive disease requiring 2 cycles of palliative 5-Fluorouracil (600 mg/m^2^/d) for 5 days every 2 weeks. The patient progressed and died of disease 1 month later. Alternatively, Wollina et al. [13] described 2 CRs and 2 PRs in 4 patients with advanced cutaneous SCC after treatment with combined chemo-immune therapy consisting of Capecitabine (950 mg/m^2^ on days 1 - 13 and subcutaneous interferon-α-2a 3 x 3 million units 3 times weekly for 3 cycles).

To the authors’ knowledge the present reports are the first describing the use of Cisplatin and Docetaxel in the management of locally advanced or metastatic cutaneous SCC. The rationale for their use was similar to that employed within other reported series; the agents have proven efficacy for SCC in other disease sites [14], they are convenient and very well tolerated, and given the impressive bulk of initial disease seen in these two cases, a powerful induction regimen was desired.
